# Bad Tumors Made Worse: SPINK1

**DOI:** 10.3389/fcell.2019.00010

**Published:** 2019-02-04

**Authors:** Christine Mehner, Evette S. Radisky

**Affiliations:** ^1^Mayo Clinic Graduate School of Biomedical Sciences, Rochester, MN, United States; ^2^Department of Cancer Biology, Mayo Clinic Comprehensive Cancer Center, Jacksonville, FL, United States

**Keywords:** SPINK1, protease inhibitor, serine protease, EGFR signaling, anoikis resistance, apoptosis resistance, chemoresistance, metastasis

## Introduction

**S**erine **p**rotease **in**hibitor **K**azal type **1** (SPINK1) is a small secreted protein with dual roles—in the pancreas, it is a protective trypsin inhibitor, while in the context of the tumor microenvironment, it is a cell growth and survival factor that promotes tumor progression. While the mechanism by which SPINK1 protects the pancreas is long established and well-understood, the mechanisms that underlie its tumor promoting properties are complex and multifaceted, with major questions remaining to be answered. In this *Opinion* article, we briefly overview the known functions and mechanisms of SPINK1 both in health and in disease, and then seek to highlight several of the mechanistic “missing links,” with the aim of identifying research opportunities and stimulating new lines of investigation.

## SPINK1—Protector of the Healthy Pancreas

**Figure 1:**
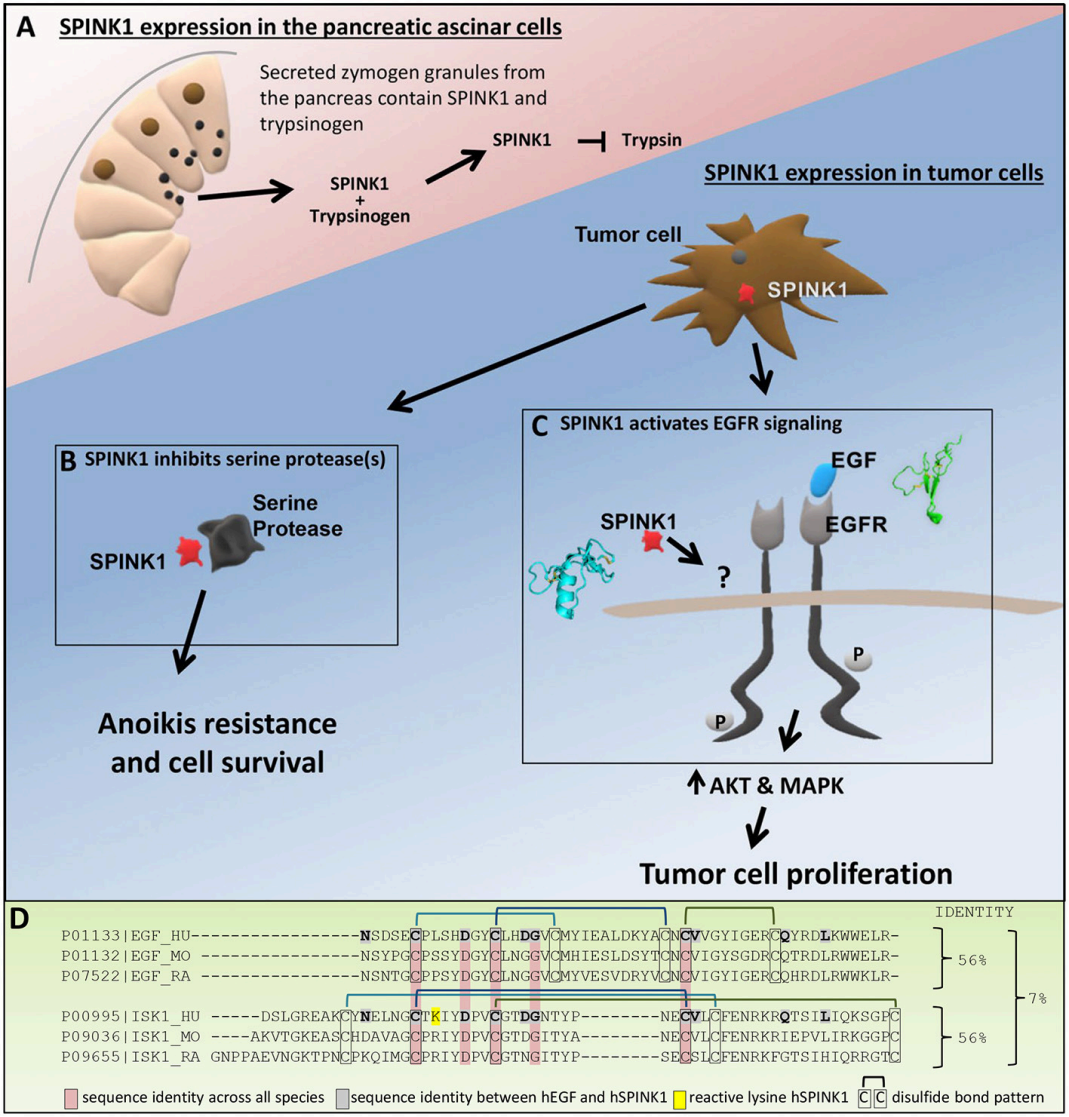
Roles of SPINK1. **(A)** In the pancreas, SPINK1 acts as an important regulator of protease activity. SPINK1 is co-expressed with trypsinogen by the pancreatic acinar cells and secreted from zymogen granules into the pancreatic duct. Within the acinar cells or the duct, SPINK1 quenches prematurely activated trypsin to prevent further protease activation and organ damage. **(B)** Tumor cell secreted SPINK1 inhibits unknown serine protease(s) to induce anoikis resistance, tumor cell survival and metastatic disease. **(C)** Tumor cell secreted SPINK1 activates EGFR kinase pathways and leads to tumor cell proliferation; the direct receptor of SPINK1 in this context requires further definition. **(D)** Sequence alignment using Clustal Omega comparing human, mouse, and rat EGF with human, mouse, and rat SPINK1 (ISK1) homologs. Identified are sequence identity between hEGF and hSPINK1, sequence identities across all three species, and disulfide bond pattern.

SPINK1, also known as pancreatic secretory trypsin inhibitor (PSTI), is a 6.2 kDa secreted serine protease inhibitor that is produced by pancreatic acinar cells. In the pancreas, SPINK1 plays a physiological role as an inhibitor of digestive trypsins (Figure 1A) (Rinderknecht, 1986; Paju and Stenman, 2006). It is co-secreted in zymogen granules with trypsinogen, the trypsin precursor protein, allowing inhibitory intervention in case of early activation of trypsinogen to trypsin, and preventing organ damage of the pancreas or duct system due to autodigestion. The importance of SPINK1 for pancreatic health is demonstrated by the association of SPINK1 gene mutations (N34S, P55S, IVS3 + 2TC, and others) with increased risk for several forms of chronic pancreatitis (Pfützer et al., 2000; Witt et al., 2000; Raphael and Willingham, 2016). Most pathogenic SPINK1 mutations reduce function of the protein by interfering with folding and/or secretion (Kiraly et al., 2007a,b; Kereszturi et al., 2009), while the N34S mutation does not appear intrinsically deleterious, but is associated with another mutation in the 5′ regulatory region of the gene that can diminish mRNA expression (Kereszturi and Sahin-Toth, 2017). On the other hand, homozygous mutations causing complete loss of SPINK1 function were found to be responsible for several cases of severe early-onset exocrine pancreatic insufficiency (Venet et al., 2017).

## SPINK1—Contributor to Poor Cancer Prognosis

Outside of the normal pancreas, aberrant expression of SPINK1 plays a role in cancer. SPINK1 was originally named tumor associated tissue inhibitor (TATI) when it was first isolated from the urine of ovarian cancer patients (Huhtala et al., 1982). Since then SPINK1 has been found to be overexpressed by multiple types of tumor cells, including breast, ovarian, prostate, pancreas, liver, and colon (reviewed Itkonen and Stenman, 2014; Rasanen et al., 2016). More recently, SPINK1 has also been found to be expressed by the tumor stroma after chemotherapy, where it may contribute to chemoresistance and increased risk of recurrence (Chen et al., 2018). SPINK1 tumor cell expression and possible prognostic value have been most studied in prostate cancer, where SPINK1 positive tumors form a subgroup of about 10–15% of prostate cancers (Tomlins et al., 2008; Ateeq et al., 2011, 2015). Prostate tumors that express SPINK1 have been reported to show a significantly more aggressive phenotype and poorer progression-free survival (Tomlins et al., 2008; Leinonen et al., 2010). In other tumor types, multiple studies have explored the potential utility of SPINK1 expression as a biomarker through analysis of tumor tissues, urine, and serum (Halila et al., 1988; Inaudi et al., 1991; de Bruijn et al., 1993; Paju et al., 2007). Tumor tissue staining for SPINK1 has been associated with poorer survival in non-serous ovarian cancers (Mehner et al., 2015) and in estrogen receptor- positive breast cancer (Soon et al., 2011), and there is potential for SPINK1 to serve as a diagnostic marker for hepatocellular carcinoma (Marshall et al., 2013). Studies in experimental model systems have demonstrated significant effects of SPINK1 in promoting tumor cell growth and survival (Rasanen et al., 2016), the mechanisms of which remain to be fully elucidated. Unlike in the normal pancreas, in tumors SPINK1 appears to be expressed independently of trypsin, and little is known about the direct target(s) of SPINK1 in the context of cancer.

## Pathogenic Functions—Resistance to Apoptotic Cell Death

Normal epithelial cells require contact to other cells or the extracellular matrix to ensure their function and survival; if they detach, intracellular mechanisms drive the apoptosis protocol called anoikis resulting in cell death. Tumor cell metastasis often involves circulation as isolated cells, and thus anoikis resistance is believed to be a common feature of metastatic dissemination (Frisch and Francis, 1994; Simpson et al., 2008; Kim et al., 2012). We have shown that SPINK1 plays an essential role in ovarian cancer cell survival under attachment free conditions (Mehner et al., 2015). Non-adherent cell survival was increased in a dose-dependent manner when treating ovarian cancer cell lines with recombinant SPINK1 protein. Notably, this effect could be mimicked by several alternative trypsin inhibitors, suggesting that anoikis resistance is mediated through the serine protease inhibitory activity of SPINK1 (Mehner et al., 2015).

SPINK1 has also been reported to confer apoptotic resistance on tumor cells in the context of chemotherapeutic treatment. Soon et al. found that SPINK1 knockdown activated apoptotic pathways in breast cancer cells, while SPINK1 overexpression induced resistance to apoptosis in cells treated with a variety of cytotoxic chemotherapy agents (Soon et al., 2011). Chemoresistance was not similarly induced by a mutant form of SPINK1 lacking the reactive site lysine residue that is required for trypsin inhibition, again implicating the serine protease inhibitory function of SPINK1 in its antiapoptotic function (Soon et al., 2011).

While evidence points to serine protease inhibition as a mechanism by which SPINK1 promotes resistance to both anoikis (Mehner et al., 2015) and chemically induced apoptosis (Soon et al., 2011) (Figure 1B), the specific serine protease target(s) of SPINK1 through which these effects are mediated are not known. The relevant apoptosis-promoting protease is unlikely to be trypsin-1 or-2, the natural physiological targets of SPINK1 in the pancreas (Rinderknecht, 1986), because although these enzymes are expressed by many tumors, they possess pro-tumorigenic activities and are predominantly associated with increased malignancy and poorer patient outcomes (Koivunen et al., 1990; Ohta et al., 1994; Yamamoto et al., 2003; Yamashita et al., 2003; Paju et al., 2004; Nyberg et al., 2006; Soreide et al., 2006). By contrast, the relevant target of SPINK1 antiapoptotic activity is expected to possess predominantly antitumor activity and to correlate with better prognosis. Besides trypsins, the human proteome includes around 80 other serine proteases with trypsin-like specificity, representing possible alternative targets for SPINK1 through which apoptosis may be regulated. Efforts to identify the SPINK1 target(s) and signaling pathways of interest could lead to identification of new biomarkers and novel points of intervention to reduce tumor cell survival and prevent spread of metastatic disease.

## Pathogenic Functions—Increased Tumor Cell Proliferation

A second important mechanism by which SPINK1 influences tumor progression is its ability to stimulate tumor cell proliferation (Rasanen et al., 2016). Here, evidence suggests that SPINK1 activates epidermal growth factor receptor (EGFR) signaling pathways (Ogawa et al., 1985; Ozaki et al., 2009; Ateeq et al., 2011; Wang et al., 2014; Mehner et al., 2015; Chen et al., 2018). In our own work we find phosphorylation of the intracellular domain of EGFR as well as phosphorylation of AKT and ERK upon treatment of ovarian cancer cells with SPINK1, consistent with activation of EGFR downstream pathways (Mehner et al., 2015). Furthermore, treatment of ovarian cancer cells with erlotinib, a selective inhibitor of the EGFR kinase domain, completely blocked the proliferative response of the cells to SPINK1, demonstrating that EGFR signaling is required for SPINK1-stimulated proliferation (Mehner et al., 2015). Others have seen similar downstream signaling of SPINK1 through EGFR in pancreatic (Ozaki et al., 2009; Wang et al., 2014), prostate (Ateeq et al., 2011), and colorectal cancers (Chen et al., 2015) (Figure 1C). SPINK1-treated pancreatic cancer cells showed increased phosphorylation of EGFR as well as activation of MAPK and STAT3; this response was attenuated in cells treated with the EGFR inhibitor AG1478 (Ozaki et al., 2009). Ateeq et al. showed in prostate cancer cells that SPINK1 knockdown reduced proliferation, which could be restored by recombinant SPINK1 protein; silencing of EGFR resulted in a significant reduction in the pro-proliferative effects of SPINK1 on the cells (Ateeq et al., 2011).

Despite the strong evidence that EGFR signaling is stimulated downstream of SPINK1, the details of how SPINK1 elicits this response remain in question. Early work by Hunt et al. (1974) identified possible sequence homology between SPINK1 and epidermal growth factor (EGF), the preferred ligand of EGFR. The possibility of functional overlap between these proteins was further suggested by studies showing that a rat SPINK1 homolog, monitor peptide, can stimulate growth of murine 3T3 fibroblasts (Fukuoka et al., 1986), and can compete with mouse EGF for binding to EGFR on the surface of these cells (Fukuoka et al., 1987). Ateeq et al. later hypothesized that human cancer cell-secreted SPINK1 may bind directly to EGFR as an alternative ligand to stimulate proliferation (Ateeq et al., 2011). Consistent with this possibility, exogenous SPINK1-GST was co-immunoprecipitated with EGFR from cell lysates (Ateeq et al., 2011), and immobilized SPINK1 showed evidence of binding to the EGFR ectodomain in a quartz-crystal microbalance assay (Ozaki et al., 2009). However, the original premise of homology between SPINK1 and EGF was based on very limited similarity between short partial sequences (Hunt et al., 1974; Scheving, 1983); only 10/56 amino acids of hSPINK1 are identical with hEGF, five of which are not conserved across species (Figure 1D). Furthermore, while SPINK1 and EGF each contain six cysteines comprising three disulfide bonds, comparison of their structures reveals entirely dissimilar protein folds (Bolognesi et al., 1982; Ogiso et al., 2002; Ferguson et al., 2003) (Figure 1C) and disulfide bonding patterns (Figure 1D). The few identical residues do not occur in similar structural contexts in the two protein families, nor do they present comparable potential binding epitopes. Thus, it is not clear why EGFR would be a natural binding target for SPINK1, and the mode of their potential interaction remains a mystery. Until stronger evidence emerges to validate and structurally characterize this binding interaction, the possible involvement of other accessory proteins or alternative SPINK1 receptors with crosstalk to EGFR should be considered (Figure 1C). For example, an earlier study by Niinobu et al. (1990) showed binding of SPINK1 to a cell surface receptor of 140 kDa, considerably smaller than EGFR, in a manner that was not diminished by competing EGF. We suggest that efforts to more clearly confirm or identify the direct receptor of SPINK1, and the mechanism by which it influences EGFR signaling, could lead to identification of novel points for therapeutic intervention in cancers that express SPINK1.

## Conclusion—A Call for New Mechanistic Studies

SPINK1 is an important contributor to both increased proliferation and metastasis development in a variety of cancers. Patients with tumors expressing SPINK1 face a poorer overall prognosis and stimulation of SPINK1 expression in the treatment-damaged tumor microenvironment may further contribute to chemoresistance and tumor recurrence. While patient studies have provided strong evidence for the importance of SPINK1 across different tumor types, the regulatory pathways that control SPINK1 expression and the direct targets of SPINK1 in the context of the tumor microenvironment, including both protease target(s) and cell surface receptor(s), remain largely unknown. The identification of specific protease targets of SPINK1 inhibition will reveal pathways controlling anoikis resistance and aid in development of biomarkers and therapeutic strategies to reduce tumor metastasis. To better understand and target SPINK1 driven tumor cell proliferation we need to further investigate the missing link between SPINK1 and EGFR signaling using modern methods and technologies. Concerted efforts are needed to uncover SPINK1 targets, signaling mechanisms and mediators, and such efforts may lead to the development of novel therapeutic strategies to reduce the impact of SPINK1 on tumors and improve patient prognosis.

## Author Contributions

All authors listed have made a substantial, direct and intellectual contribution to the work, and approved it for publication.

### Conflict of Interest Statement

The authors declare that the research was conducted in the absence of any commercial or financial relationships that could be construed as a potential conflict of interest.

## References

[B1] Ateeq B. Kunju L. P. Carskadon S. L. Pandey S. K. Singh G. Pradeep I.. (2015). Molecular profiling of ETS and non-ETS aberrations in prostate cancer patients from northern India. Prostate 75, 1051–1062. 10.1002/pros.22989 25809148 PMC4832366

[B2] Ateeq B. Tomlins S. A. Laxman B. Asangani I. A. Cao Q. Cao X.. (2011). Therapeutic targeting of SPINK1-positive prostate cancer. Sci. Transl. Med. 3:72ra17. 10.1126/scitranslmed.3001498 21368222 PMC3211047

[B3] Bolognesi M. Gatti G. Menagatti E. Guarneri M. Marquart M. Papamokos E.. (1982). Three-dimensional structure of the complex between pancreatic secretory trypsin inhibitor (Kazal type) and trypsinogen at 1.8 A resolution. Structure solution, crystallographic refinement and preliminary structural interpretation. J. Mol. Biol. 162, 839–868. 10.1016/0022-2836(82)90550-2 7169635

[B4] Chen F. Long Q. Fu D. Zhu D. Ji Y. Han L.. (2018). Targeting SPINK1 in the damaged tumour microenvironment alleviates therapeutic resistance. Nat. Commun. 9:4315. 10.1038/s41467-018-06860-4 30333494 PMC6193001

[B5] Chen Y. T. Tsao S. C. Yuan S. S. Tsai H. P. Chai C. Y. (2015). Serine protease inhibitor Kazal type 1 (SPINK1) promotes proliferation of colorectal cancer through the epidermal growth factor as a prognostic marker. Pathol. Oncol. Res. 21, 1201–1208. 10.1007/s12253-015-9949-0 26037168

[B6] de Bruijn H. W. ten Hoor K. A. Boonstra H. Marrink J. Krans M. Aalders J. G. (1993). Cancer-associated antigen CA 195 in patients with mucinous ovarian tumours: a comparative analysis with CEA, TATI and CA 125 in serum specimens and cyst fluids. Tumour Biol. 14, 105–115. 10.1159/000217863 8392219

[B7] Ferguson K. M. Berger M. B. Mendrola J. M. Cho H. S. Leahy D. J. Lemmon M. A. (2003). EGF activates its receptor by removing interactions that autoinhibit ectodomain dimerization. Mol. Cell 11, 507–517. 10.1016/S1097-2765(03)00047-9 12620237

[B8] Frisch S. M. Francis H. (1994). Disruption of epithelial cell-matrix interactions induces apoptosis. J. Cell Biol. 124, 619–626. 10.1083/jcb.124.4.619 8106557 PMC2119917

[B9] Fukuoka S. Fushiki T. Kitagawa Y. Sugimoto E. Iwai K. (1986). Growth stimulating activity on 3T3 fibroblasts of the molecular weight 6,500-peptide purified from rat pancreatic juice. Biochem. Biophys. Res. Commun. 139, 545–550. 10.1016/S0006-291X(86)80025-0 3767976

[B10] Fukuoka S. Fushiki T. Kitagawa Y. Sugimoto E. Iwai K. (1987). Competition of a growth stimulating-/cholecystokinin (CCK) releasing-peptide (monitor peptide) with epidermal growth factor for binding to 3T3 fibroblasts. Biochem. Biophys. Res. Commun. 145, 646–650. 10.1016/0006-291X(87)91013-8 3496093

[B11] Halila H. Lehtovirta P. Stenman U. H. (1988). Tumour-associated trypsin inhibitor (TATI) in ovarian cancer. Br. J. Cancer 57, 304–307. 10.1038/bjc.1988.67 3162682 PMC2246526

[B12] Huhtala M. L. Pesonen K. Kalkkinen N. Stenman U. H. (1982). Purification and characterization of a tumor-associated trypsin inhibitor from the urine of a patient with ovarian cancer. J. Biol. Chem. 257, 13713–13716. 7142173

[B13] Hunt L. T. Barker W. C. Dayhoff M. O. (1974). Epidermal growth factor: Internal duplication and probable relationship to pancreatic secretory trypsin inhibitor. Biochem. Biophys. Res. Commun. 60, 1020–1028. 10.1016/0006-291X(74)90415-X 4429557

[B14] Inaudi P. Petrilli S. De Leo V. Bernabei A. Pasqui L. D'Antona N. (1991). Evaluation of tumor-associated trypsin inhibitor (TATI) in women with benign and malignant gynecological disease. Scand. J. Clin. Lab. Invest. Suppl. 207, 11–13. 10.3109/00365519109104619 1780683

[B15] Itkonen O. Stenman U. H. (2014). TATI as a biomarker. Clin. Chim. Acta 431, 260–269. 10.1016/j.cca.2014.02.014 24583226

[B16] Kereszturi E. Kiraly O. Sahin-Toth M. (2009). Minigene analysis of intronic variants in common SPINK1 haplotypes associated with chronic pancreatitis. Gut 58, 545–549. 10.1136/gut.2008.164947 18978175 PMC2677899

[B17] Kereszturi E. Sahin-Toth M. (2017). Pancreatic cancer cell lines heterozygous for the SPINK1 p.N34S haplotype exhibit diminished expression of the variant allele. Pancreas 46, e54–e55. 10.1097/mpa.0000000000000817 28609377 PMC5470582

[B18] Kim Y.-N. Koo K. H. Sung J. Y. Yun U.-J. Kim H. (2012). Anoikis resistance: an essential prerequisite for tumor metastasis. Int. J. Cell Biol. 2012:306879. 10.1155/2012/306879 22505926 PMC3296207

[B19] Kiraly O. Boulling A. Witt H. Le Marechal C. Chen J. M. Rosendahl J.. (2007a). Signal peptide variants that impair secretion of pancreatic secretory trypsin inhibitor (SPINK1) cause autosomal dominant hereditary pancreatitis. Hum. Mutat. 28, 469–476. 10.1002/humu.20471 17274009 PMC2765331

[B20] Kiraly O. Wartmann T. Sahin-Toth M. (2007b). Missense mutations in pancreatic secretory trypsin inhibitor (SPINK1) cause intracellular retention and degradation. Gut 56, 1433–1438. 10.1136/gut.2006.115725 17525091 PMC2000263

[B21] Koivunen E. Itkonen O. Halila H. Stenman U. H. (1990). Cyst fluid of ovarian cancer patients contains high concentrations of trypsinogen-2. Cancer Res. 50, 2375–2378. 2180568

[B22] Leinonen K. A. Tolonen T. T. Bracken H. Stenman U. H. Tammela T. L. Saramaki O. R.. (2010). Association of SPINK1 expression and TMPRSS2:ERG fusion with prognosis in endocrine-treated prostate cancer. Clin. Cancer Res. 16, 2845–2851. 10.1158/1078-0432.ccr-09-2505 20442300

[B23] Marshall A. Lukk M. Kutter C. Davies S. Alexander G. Odom D. T. (2013). Global gene expression profiling reveals SPINK1 as a potential hepatocellular carcinoma marker. PLoS ONE 8:e59459. 10.1371/journal.pone.0059459 23527199 PMC3601070

[B24] Mehner C. Oberg A. L. Kalli K. R. Nassar A. Hockla A. Pendlebury D.. (2015). Serine protease inhibitor Kazal type 1 (SPINK1) drives proliferation and anoikis resistance in a subset of ovarian cancers. Oncotarget 6, 35737–35754. 10.18632/oncotarget.5927 26437224 PMC4742138

[B25] Niinobu T. Ogawa M. Murata A. Nishijima J. Mori T. (1990). Identification and characterization of receptors specific for human pancreatic secretory trypsin inhibitor. J. Exp. Med. 172, 1133–1142. 10.1084/jem.172.4.1133 2170560 PMC2188617

[B26] Nyberg P. Ylipalosaari M. Sorsa T. Salo T. (2006). Trypsins and their role in carcinoma growth. Exp. Cell Res. 312, 1219–1228. 10.1016/j.yexcr.2005.12.024 16457812

[B27] Ogawa M. Tsushima T. Ohba Y. Ogawa N. Tanaka S. Ishida M.. (1985). Stimulation of DNA synthesis in human fibroblasts by human pancreatic secretory trypsin inhibitor. Res. Commun. Chem. Pathol. Pharmacol. 50, 155–158. 3909270

[B28] Ogiso H. Ishitani R. Nureki O. Fukai S. Yamanaka M. Kim J. H.. (2002). Crystal structure of the complex of human epidermal growth factor and receptor extracellular domains. Cell 110, 775–787. 10.1016/S0092-8674(02)00963-7 12297050

[B29] Ohta T. Terada T. Nagakawa T. Tajima H. Itoh H. Fonseca L.. (1994). Pancreatic trypsinogen and cathepsin B in human pancreatic carcinomas and associated metastatic lesions. Br. J. Cancer 69, 152–156. 10.1038/bjc.1994.25 8286198 PMC1968761

[B30] Ozaki N. Ohmuraya M. Hirota M. Ida S. Wang J. Takamori H.. (2009). Serine protease inhibitor Kazal type 1 promotes proliferation of pancreatic cancer cells through the epidermal growth factor receptor. Mol. Cancer Res. 7, 1572–1581. 10.1158/1541-7786.mcr-08-0567 19737965

[B31] Paju A. Hotakainen K. Cao Y. Laurila T. Gadaleanu V. Hemminki A.. (2007). Increased expression of tumor-associated trypsin inhibitor, TATI, in prostate cancer and in androgen-independent 22Rv1 cells. Eur. Urol. 52, 1670–1679. 10.1016/j.eururo.2007.01.096 17306443

[B32] Paju A. Stenman U. H. (2006). Biochemistry and clinical role of trypsinogens and pancreatic secretory trypsin inhibitor. Crit. Rev. Clin. Lab. Sci. 43, 103–142. 10.1080/10408360500523852 16517420

[B33] Paju A. Vartiainen J. Haglund C. Itkonen O. von Boguslawski K. Leminen A.. (2004). Expression of trypsinogen-1, trypsinogen-2, and tumor-associated trypsin inhibitor in ovarian cancer: prognostic study on tissue and serum. Clin. Cancer Res. 10, 4761–4768. 10.1158/1078-0432.CCR-0204-03 10/14/4761 15269150

[B34] Pfützer R. H. Barmada M. M. Brunskill A. P. Finch R. Hart P. S. Neoptolemos J.. (2000). SPINK1/PSTI polymorphisms act as disease modifiers in familial and idiopathic chronic pancreatitis. Gastroenterology 119, 615–623. 10.1053/gast.2000.18017 10982753

[B35] Raphael K. L. Willingham F. F. (2016). Hereditary pancreatitis: current perspectives. Clin. Exp. Gastroenterol. 9, 197–207. 10.2147/ceg.s84358 27555793 PMC4968666

[B36] Rasanen K. Itkonen O. Koistinen H. Stenman U. H. (2016). Emerging roles of SPINK1 in cancer. Clin. Chem. 62, 449–457. 10.1373/clinchem.2015.241513 26656134

[B37] Rinderknecht H. (1986). Activation of pancreatic zymogens. Normal activation, premature intrapancreatic activation, protective mechanisms against inappropriate activation. Dig. Dis. Sci. 31, 314–321. 10.1007/BF01318124 2936587

[B38] Scheving L. A. (1983). Primary amino acid sequence similarity between human epidermal growth factor-urogastrone, human pancreatic secretory trypsin inhibitor, and members of porcine secretin family. Arch. Biochem. Biophys. 226, 411–413. 10.1016/0003-9861(83)90309-0 6605724

[B39] Simpson C. D. Anyiwe K. Schimmer A. D. (2008). Anoikis resistance and tumor metastasis. Cancer Lett. 272, 177–185. 10.1016/j.canlet.2008.05.029 18579285

[B40] Soon W. W. Miller L. D. Black M. A. Dalmasso C. Chan X. B. Pang B.. (2011). Combined genomic and phenotype screening reveals secretory factor SPINK1 as an invasion and survival factor associated with patient prognosis in breast cancer. EMBO Mol. Med. 3, 451–464. 10.1002/emmm.201100150 21656687 PMC3377086

[B41] Soreide K. Janssen E. Körner H. Baak J. (2006). Trypsin in colorectal cancer: molecular biological mechanisms of proliferation, invasion, and metastasis. J. Pathol. 209, 147–156. 10.1002/path.1999 16691544

[B42] Tomlins S. A. Rhodes D. R. Yu J. Varambally S. Mehra R. Perner S.. (2008). The role of SPINK1 in ETS rearrangement-negative prostate cancers. Cancer Cell 13, 519–528. 10.1016/j.ccr.2008.04.016 18538735 PMC2732022

[B43] Venet T. Masson E. Talbotec C. Billiemaz K. Touraine R. Gay C.. (2017). Severe infantile isolated exocrine pancreatic insufficiency caused by the complete functional loss of the SPINK1 gene. Hum. Mutat. 38, 1660–1665. 10.1002/humu.23343 28945313

[B44] Wang C. Wang L. Su B. Lu N. Song J. Yang X.. (2014). Serine protease inhibitor Kazal type 1 promotes epithelial-mesenchymal transition through EGFR signaling pathway in prostate cancer. Prostate 74, 689–701. 10.1002/pros.22787 24619958

[B45] Witt H. Luck W. Hennies H. C. Classen M. Kage A. Lass U.. (2000). Mutations in the gene encoding the serine protease inhibitor, Kazal type 1 are associated with chronic pancreatitis. Nat. Genet. 25, 213–216. 10.1038/76088 10835640

[B46] Yamamoto H. Iku S. Adachi Y. Imsumran A. Taniguchi H. Nosho K.. (2003). Association of trypsin expression with tumour progression and matrilysin expression in human colorectal cancer. J. Pathol. 199, 176–184. 10.1002/path.1277 12533830

[B47] Yamashita K. Mimori K. Inoue H. Mori M. Sidransky D. (2003). A tumor-suppressive role for trypsin in human cancer progression. Cancer Res. 63, 6575–6578. Available online at: http://cancerres.aacrjournals.org/content/63/20/6575 14583448

